# Wnt5a regulates Ameloblastoma Cell Migration by modulating Mitochondrial and Cytoskeletal Dynamics

**DOI:** 10.7150/jca.46547

**Published:** 2020-07-11

**Authors:** Xue Qiao, Xing Niu, Junxiu Shi, Lijie Chen, Xiaobin Wang, Jinwen Liu, Li Zhu, Ming Zhong

**Affiliations:** 1 Department of Oral Histopathology, School and Hospital of Stomatology, China Medical University, Liaoning Province Key Laboratory of Oral Disease, Shenyang, Liaoning, China.; 2 Department of Stomatology, Xiang'an Hospital of Xiamen University, Xiamen, Fujian, China.; 3 Department of Central Laboratory, School and Hospital of Stomatology, China Medical University, Liaoning Province Key Laboratory of Oral Disease, Shenyang, Liaoning, China.; 4 Department of Oral Biology, School and Hospital of Stomatology, China Medical University, Liaoning Province Key Laboratory of Oral Disease, Shenyang, Liaoning, China.; 5 Department of Developmental Cell Biology, Cell Biology Division, Key Laboratory of Cell Biology, Ministry of Public Health, Key Laboratory of Medical Cell Biology, Ministry of Education, China Medical University, Shenyang, Liaoning, China.; 6 Department of Orthodontics, School and Hospital of Stomatology, China Medical University, Liaoning Province Key Laboratory of Oral Disease, Shenyang, Liaoning, China.; 7 Department of Periodontics, School and Hospital of Stomatology, China Medical University, Liaoning Province Key Laboratory of Oral Disease, Shenyang, Liaoning, China.

**Keywords:** ameloblastoma, mitochondria-cytoskeleton, Wnt5a, Coro1A, migration

## Abstract

**Objective:** Abnormal expression of Wnt5a has been detected in various tumors, including ameloblastoma (AB). Yet, there is no specific mechanistic evidence for the functional role of Wnt5a in AB. In this study, we aimed to conduct a mechanistic examination of the importance of Wnt5a in AB development.

**Methods:** The expressions of Wnt5a and Coro1A were examined by Western blot and immunohistochemistry both in AB tissues and AM-1 cells. The number and size of mitochondria were detected by electronic transmission microscope and confocal microscope. Gain-of-function and loss-of-function assays were used to explore the biological roles of Wnt5a and Coro1A in organelle dynamics changes and cell migration. Cell migration was detected by wound healing and transwell assay.

**Results:** We found that in AM-1 cells, up-regulation of Wnt5a led to enhanced mitochondrial energy production and altered calcium homeostasis, with elevated calcium levels directly leading to altered mitochondrial dynamics and interactions between the cytoskeleton and the mitochondria. When Wnt5a or its downstream cytoskeleton-associated protein Coro1A was knocked down, the migration capacity of AM-1 cells was markedly impaired.

**Conclusion:** Together, these results suggest that Wnt5a plays mitochondria and cytoskeleton specific roles in regulating the development of human AB, with its down-regulation leading to impaired tumor development, thus highlighting Wnt5a or Coro1A as potentially viable therapeutic targets for the treatment of AB.

## Introduction

Ameloblastoma (AB) is a common epithelial tumor, accounting for more than 60% of odontogenic tumors 1, 2. AB is typically composed of enamel-like structures without any mature enamel or hard tissue being present. According to the latest WHO Classification of Head and Neck Tumors, ABs are highly diverse with four primary pathological subtypes being recognized: AB, unicystic, and extraosseous/peripheral types 3. These tumors typically arise in the jaw, driving localized swelling and deformities of the face 4. Typical treatment of ABs entails radical jaw excision, but the resultant facial deformities could have a markedly adverse impact on the physical and mental health of treated patients. However, when patients instead undergo more conservative treatment, recurrence is common, in some cases leading to malignant transformation and metastasis 5-7. As such, it is vital that molecular therapeutic targets are identified to guide AB treatment so as to ensure that patients have satisfactory clinical outcomes.

Mitochondria are essential intracellular organelles both for regulating energy production within cells, and for buffering intracellular Ca^2+^ levels and mediating interactions between organelles. It is also well known that they are closely linked with the development of tumors, with mitochondrial damage in tumor cells disrupting the normal balance between oxidative phosphorylation and glycolysis, thereby resulting in characteristic metabolic reorganization that is frequently observed in tumors 8, 9. The number, morphology, and localization of mitochondria within cells are highly variable, and are closely related to the invasive and migratory capabilities of tumor cells 10, 11. The cytoskeleton can also regulate mitochondrial intracellular dynamics. Some studies suggest that actin-related proteins regulate mitochondrial fission and contact between mitochondria and the cytoskeleton 12. Remodeling of the cytoskeleton and mitochondrial network can have a profound impact on the motility of cells, and is thus a key component of tumor progression 13. However, to date, no studies have specifically examined the changes in mitochondrial dynamics or organelle interactions that occur during AB development, with the underlying molecular mechanisms therefore being wholly uncharacterized.

Proteins in the Wnt family facilitate paracrine and autocrine activation of specific cell membrane receptors 14. Wnt5a can regulate cellular signaling through non-canonical Wnt signaling pathways, with reported roles in the development and progression of various tumor types 15, including elevated Wnt5a expression in oral squamous cell carcinoma, tongue cancer and ameloblastoma 16-18. Wnt family proteins have been reported to play key roles in regulation of mitochondrial quality control and energy metabolism. For example, Wnt3a overexpression mediates enhanced mitochondrial basal oxygen consumption and up-regulates proteins associated with oxidative phosphorylation 19. Classical Wnt/β-catenin signaling can, in concert with PTEN signaling, additionally mediate the enhanced fusion of damaged mitochondria and inhibit mitophagy, resulting in altered mitochondrial remodeling, abnormal mitochondrial accumulation, and altered cellular migration and motility 20. How the non-canonical Wnt5a/Ca^2+^ signaling pathway regulates mitochondrial network dynamics and organelle interactions within cells, however, is not as well understood.

In the present study, we aimed to expound the specific mechanistic evidence for the functional role of up-regulated Wnt5a in AB. Its overexpression led to significant increases in mitochondrial and intracellular calcium, resulting in substantial mitochondrial and cytoskeletal remodeling. When Wnt5a or its downstream cytoskeleton associated target protein Coro1A were knocked down, this significantly ablated these changes in intracellular organelle dynamics and suppressed the migratory activity of AB cells. At present, there is a lack of relevant research on the role of Coro1A in AB. In summary, these findings offer a novel insight into the function of Wnt5a in the regulation of organelle dynamics and AB progression, potentially highlighting this protein and its associated pathway as viable targets for AB therapy.

## Materials and Methods

### Tissue specimens

In total, 15 paired AB and adjacent normal tissue specimens were obtained from patients that underwent surgical tumor resection at the Department of Oral and Maxillofacial Surgery in the School of Stomatology of China Medical University from 2016 to 2018. Upon collection, tissues were immediately stored at -80 °C prior to downstream analysis. We also collected 91 AB immunohistochemical sections from the School and Hospital of Stomatology, China Medical University between January 2015 to March 2016. Moreover, 20 cases of normal oral mucosa (NOM) tissues were obtained from patients undergoing surgical removal of the third mandibular molar. The study was approved by the Ethics Committee of School and Hospital of Stomatology, China Medical University (2016-12), and all patients signed informed consent.

### Cell culture

AM-1 cells used in the present study were generously gifted by Professor Hidemitsu Harada (School of Dentistry, Iwate Medical University, Japan), and were grown between passage 15 and 25 on the Keratinocyte-SFM (Gibco, Invitrogen, USA). Human keratinocytes (HaCaT cells, ATCC, USA) from passage 5-17 were cultured in DMEM (Gibco, Invitrogen, USA) plus 10% FBS and penicillin/streptomycin.

### Immunohistochemistry

After paraffin embedding, 4 μm thick sections of AB tissue samples were prepared. These sections were deparaffinized by xylene, rehydrated by an ethanol gradient, and quenched by endogenous peroxidase activity via treatment for 30 min in methanol containing 0.3% H_2_O_2_. Samples were then microwaved in citrate phosphate buffer (pH 6.0) to mediate antigen retrieval. Then, samples were incubated with polyclonal rabbit anti-Wnt5a (1:2000, ab229200, Abcam, MA, USA) at 4 °C overnight, followed by secondary anti-rabbit IgG (1:5000, Santa Cruz Biotechnology, USA) at room temperature for 2 h.

Staining results were scored based upon both (A) staining intensity (with 0, 1, 2, and 3 corresponding to negative, weak, moderate, and strong staining, respectively), and (B) Wnt5a-positive tumor cells frequency (with 1, 2, 3, and 5 corresponding to 0-10%, 11-50%, 51-80%, and 81-100%, respectively) 21. These two scores were then multiplied together, yielding a final staining score. When scores >3, the samples were considered Wnt5a-positive, otherwise they were considered Wnt5a-negative.

### Western blot

After protein extraction from the indicated samples, equivalent amounts (40-80 μg) of protein from each sample was separated via 10% SDS-PAGE and transferred onto PVDF membranes. These blots were then blocked with 5% non-fat milk and incubated at 4 °C overnight with the following antibodies: anti-Wnt5a (1:2000, ab229200, Abcam), anti-GAPDH (1:2500, ab9485, Abcam), anti-Coro1A (1:2000, ab72212, Abcam). GAPDH was used as an internal control. Blots were then incubated with secondary goat anti-rabbit IgG (1:10000, A21020, Abbkine) for 1h. Finally, Odyssey imaging system was used for protein visualization.

### Confocal microscopy

FITC-phalloidin (0.2 μM, 30 min) and Mito-Tracker Red (0.2 μM, 10 min) (Invitrogen, USA) were used to treat AM-1 cells, after which a Nikon A1+ confocal microscope equipped with a 40X 1.3NA oil immersion objective was used for the confocal imaging of these stained tumor cells. Excitation at 488 nm and 562 nm was used to mediate dual excitation of these two fluorophores, with respective emission being detected at 505-530 nm and > 560 nm, respectively.

### Assessment of mitochondrial respiration

A Seahorse XF24 Extracellular Flux Analyzer (Seahorse Bioscience, USA) was used to assess mitochondrial respiration based on provided directions. Briefly, AM-1 cells were added to XF microplates (10,000 cells/well), after which cellular OCR was assessed in assay medium (Sigma, USA) to which oligomycin (1 μM), FCCP (500 nM), rotenone (1 μM), and antimycin A (1 μM) were added in sequence.

### Intracellular calcium imaging

Initially, 5×10^4^ AM-1 cells were plated on 25 mm coverslips and were treated for 30 min with 1 μM Fluo-4 (Molecular Probes, Invitrogen, USA) at 37 °C. Cells were then washed thrice with KSFM media followed by confocal imaging with a Nikon A1+ confocal microscope (Nikon, Japan). Cells were imaged with excitation and emission wavelengths of 488 nm and 505-530 nm, respectively, with bidirectional scanning mode being used to acquire 400 128×128 pixel frames (0.244s/frame). The Wnt5a-containing media was then added to cells, and cell images were recorded for 5min, with fluorescence intensity being reported as ΔF/F_0_ as indicated by (F-F_base_)/F_base_.

### Cell migration assay

The migration of AM-1 cells was assessed using wound healing and transwell assays. For the former, a monolayer of AM-1 cells (80% confluent) was scratched using a 100 μl pipette tip, after which this wound was washed thrice using PBS and the migration distance of wound edge was calculated with Image J software. In the transwell assays, AM-1 cells in 100 μl serum-free media were added to the upper chamber of a 24-well plate (Costar, USA). After 24 h, the cells that had migrated to the lower chamber of this plate were fixed using ethanol and stained with hematoxylin and eosin.

### Recombinant adenovirus preparation

Double Wnt5a-specific siRNA sequences (5'-GAAGCCCAUUGGAAUAUUAUU-3' and 5'-GCUGGAAGUGCAAUGUCUUCC-3') were designed based upon the Wnt5a gene sequence and were synthesized by Genepharma (Shanghai, China). A non-targeted siRNA sequence 5'-UUUGCACUGUGCAAGCCUCUU-3' was used as a negative control. These sequences were inserted into the pENTR plasmid recombined with pBLOCK-iT™ 6-DEST vector and an Adenoviral Expression System (Invitrogen) was used to prepare adenoviral particles that were purified with a Vivapure®AdenoPACKTM20RT Kit (Sartorius). A Coro1A-specific siRNA (5'-UUGUCUACUCGUCCAGUCUUGCCUA-3') was also designed and synthesized. These sequences were then used to prepare into adenoviral particles as above.

### Reagents

Recombinant human Wnt5a protein was purchased from R&D systems; anti-Wnt5a was from Abcam; anti-Coro1A and anti-NFATc2 were from Affinity; anti-Lamin B1 was from Origene; Mitotracker Red, phalloidin FITC, and fluo-4 were from Molecular Probes.

### Statistical analysis

Data are presented as means ± SEM. Each experiment was repeated at least three times. The difference was compared via unpaired Student's t-test or one-way ANOVA with Bonferroni's post hoc test as appropriate. In addition, correlations between the Coro1A expression and clinical characteristics of AB patients were assessed by Chi-square test. GraphPad Prism 5.0 (Graph Pad Software, Inc., San Diego, CA, USA) was used for all statistical analyses, with P<0.05 as the significant threshold.

## Results

### AB tissues exhibit elevated Wnt5a expression

**Figure 1:**
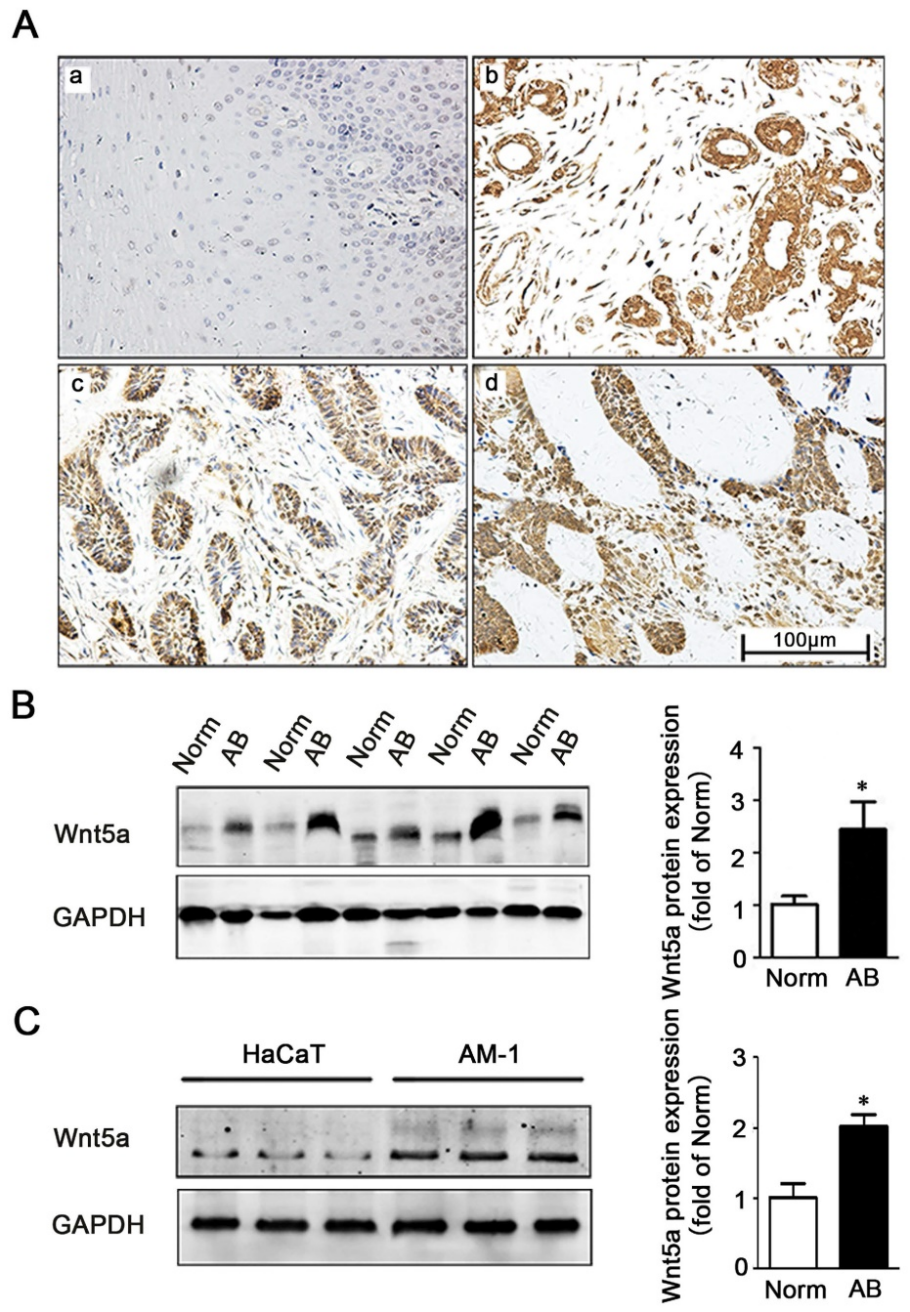
** Expression of Wnt5a in ameloblastoma tissues and AM-1 cells.** (**A**) Immunohistochemistry staining demonstrating Wnt5a protein expression in normal oral mucosa tissues (a), ameloblastoma tissues (b-d) (forlicular type for d&c, plexiform type for d). Scale Bar: 100 µm. Magnification: 200×. (**B**) Western blot showed Wnt5a protein expression in tumor adjacent normal and ameloblastoma tissues. (**C**) Western blot showed Wnt5a protein expression in HaCaT and AM-1 cell lines. *p<0.05.

We firstly explored the role of Wnt5a in AB by comparing its immunohistochemical expression in 91 AB tissues and 20 normal tissues (**Figure 1A**). Consistent with previously studies by Sukarawan et al. 18, we found that Wnt5a expression was clearly evident in AB samples, and the positive expression rate in these tumor tissues was significantly higher than normal control samples (**Table 1**). This result was further supported by Western blot, which additionally confirmed elevated Wnt5a protein expression in AB tissues (**Figure 1B**). Furthermore, we observed about 2-fold increase in Wnt5a expression in an AB cell line (AM-1 cell line) relative to levels in control HaCaT cells (**Figure 1C**). Together, these data suggest a possible involvement of Wnt5a in the process of AB development.

### Wnt5a regulates energy synthesis by modulating mitochondrial calcium homeostasis

**Figure 2:**
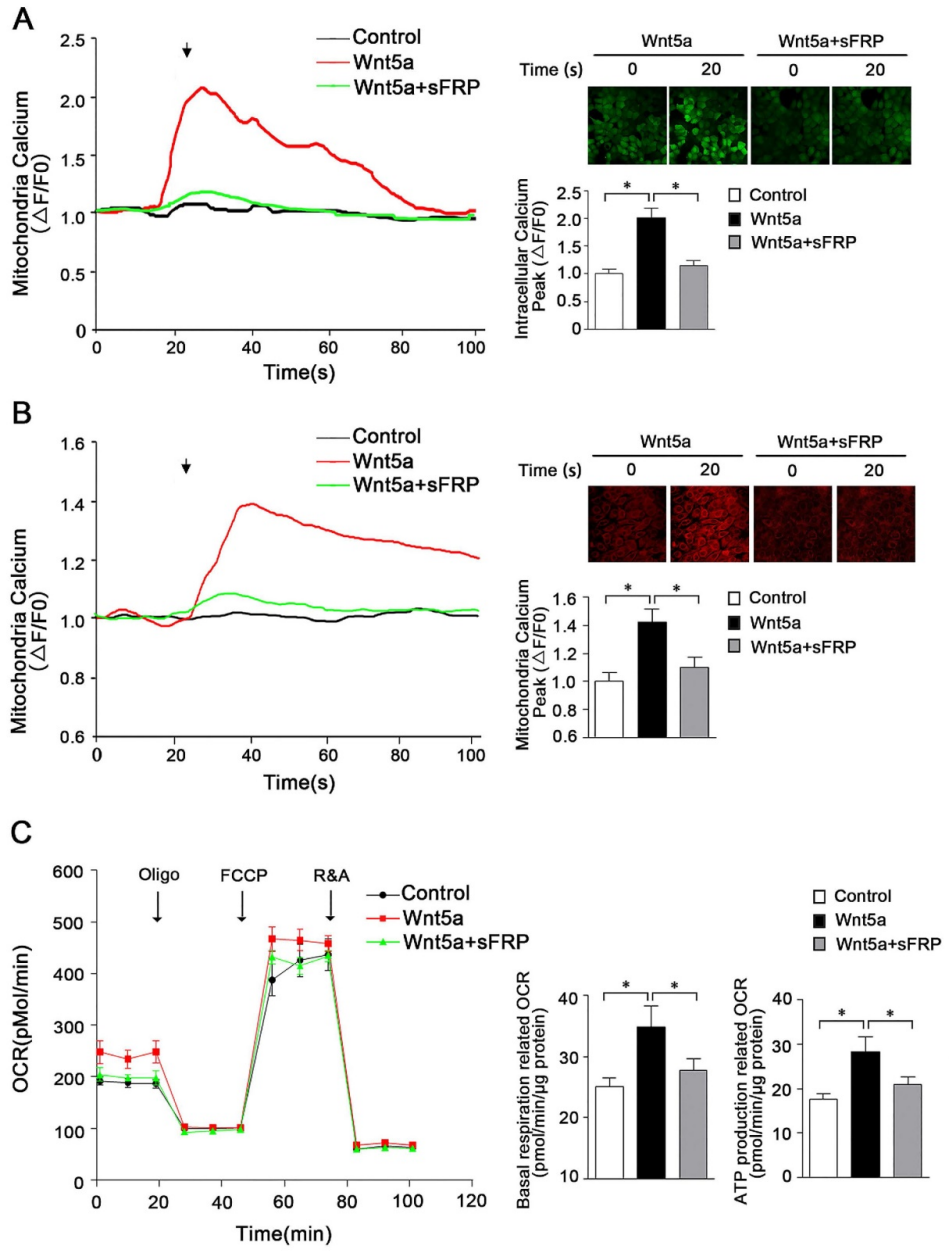
** Wnt5a regulates energy synthesis through manipulating mitochondria calcium hemostasis.** (**A**) Confocal images and representative recordings of cytosolic Ca^2+^ in Fluo-4-AM loaded AM-1 cells. The graph showing intracellular calcium changes induced by Wnt5a (200 nm) recombinant protein or Wnt5a plus sFRP5 protein addition. N=30 cells from 4 independent experiments. Bar chart represented quantification of peak amplitude. (**B**) Confocal images and representative recordings of mitochondrial Ca^2+^ in Rhod-2-AM loaded AM-1 cells. The graph showing mitochondria calcium change after Wnt5a or Wnt5a plus sFRP5 recombinant protein addition. N=32-35 cells from 4 independent experiments. Bar chart represented quantification of peak amplitude. (**C**) Traces of oxygen consumption rate (OCR) of AM-1 cells treated with Wnt5a or Wnt5a plus sFRP5, as measured with the XF24 metabolic analyzer by sequential, in port additions of mitochondrial effectors at time points indicated by downward arrows. Bar charts illustrating basal respiration related OCR and ATP production related OCR, n=10 from 3 independent experiments. *p<0.05.

Previous research has highlighted a role for secreted Wnt5a in mediating signaling that ultimately leads to the opening of endoplasmic reticulum IP_3_R, resulting in Ca^2+^ release into the cytoplasm 22, 23. However, in contrast to these reports, work from Koopmans et al. suggests that Wnt5a does not play a significant role in the regulation of calcium homeostasis in the cytoplasm 24. As such, further research was warranted to understand whether Wnt5a plays a role in regulating mitochondrial calcium homeostasis in AB cells, and if so what signaling pathways are involved in such regulatory activity. To that end, we assessed cytoplasmic and mitochondrial calcium and ATP levels in the human AM-1 cells following treatment with recombinant Wnt5a. These results revealed the ability of Wnt5a to both drive mitochondrial energy production and to modulate intracellular calcium homeostasis within these cells, with intracellular and mitochondrial peak calcium levels rising by 2.02 ± 0.16 folds and 1.41 ± 0.11 folds following Wnt5a treatment, respectively, relative to levels in control cells (**Figure 2A, B**). Treatment with Wnt5a further led to elevated basal respiration-associated OCR and ATP production-associated OCR (**Figure 2C**). When we treated cells with sFRP5, a well-recognized Wnt signaling pathway antagonist, to inhibit Wnt5a, both the cytoplasmic and mitochondria calcium increase had greatly dropped and no statistically significant increase was observed in basal respiration and ATP production associated OCR compared with control cells (**Figure 2**). Together, these findings thus suggest that in AB cells, Wnt5a can induce slight changes in calcium homeostasis that in turn alter mitochondrial function within these cells.

### Wnt5a modulates mitochondrial dynamics in ameloblastoma

**Figure 3:**
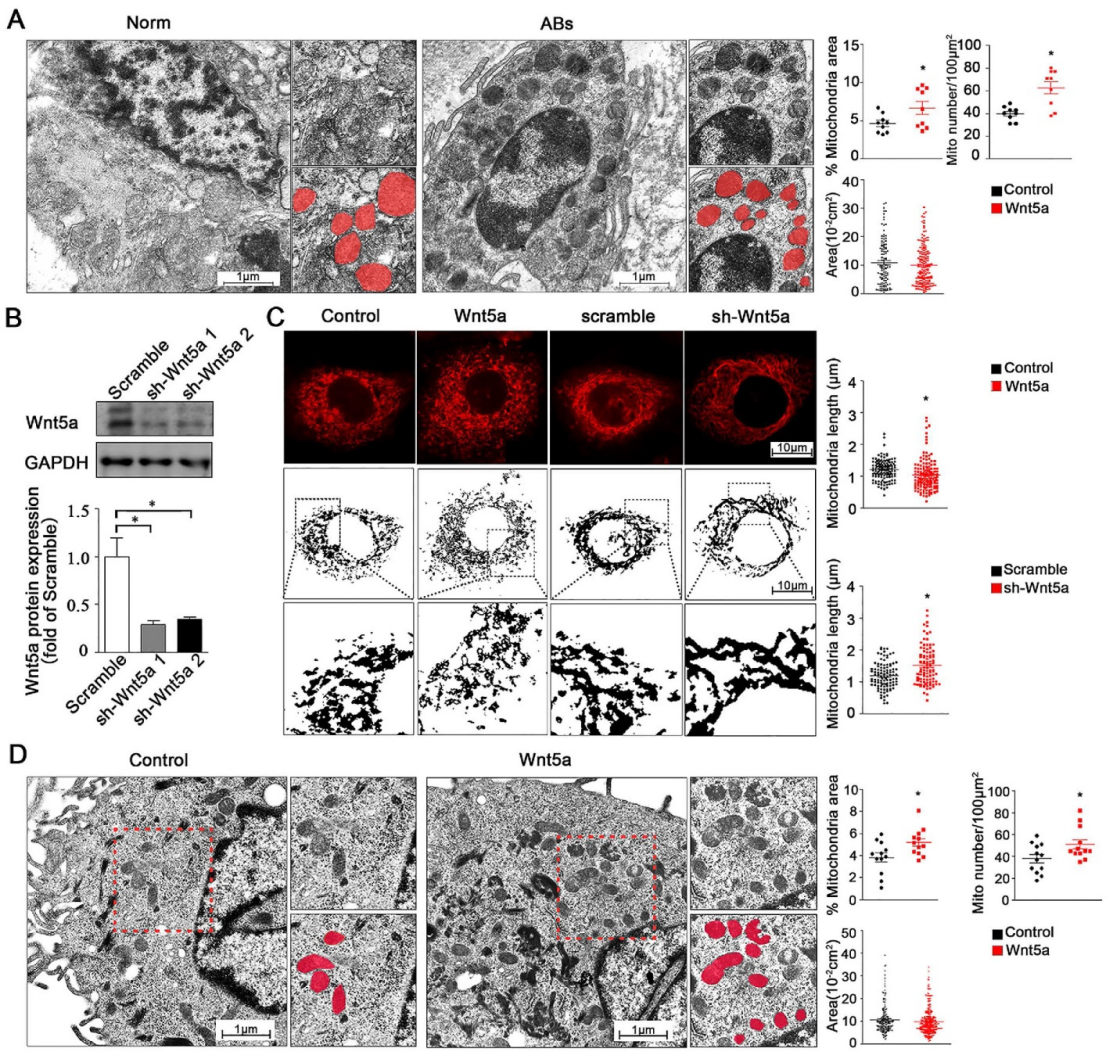
** Wnt5a regulates mitochondria dynamics and morphology.** (**A**) Micrographs from transmission electron microscope showing representative mitochondria morphology and number of tumor adjacent normal tissues and ameloblastoma tissues. Corresponding quantification plot charts demonstrating percentage mitochondria area fraction, mitochondria number per 100 µm^2^ and average area of single mitochondria. N=9 for area and number, N=114,116 independent mitochondria from 3 independent experiments for single mitochondria. (**B**) Western blot detecting protein expression manipulated by Wnt5a shRNA adenovirus. (**C**) Confocal images showing representative mitochondria morphology of AM-1 cells treated with Wnt5a recombinant protein or Wnt5a knockdown adenovirus. Scatter plot charts showed quantitative analysis of mitochondria length. N=126,145 for Wnt5a up-regulation and N=101, 94 for Wnt5a knockdown from 3 independent experiments. (**D**) TEM images demonstrated mitochondria number and size of AM-1 cells with or without Wnt5a protein addition. Corresponding quantification demonstrate percentage mitochondria area fraction, mitochondria number per 100 µm^2^ and single mitochondria area. n=12 for mitochondria area fraction and number, N=222,302 for independent mitochondria from 4 independent experiments. *p<0.05.

In addition to regulate mitochondrial energy production, changes in calcium homeostasis are also closely linked to both cellular survival and mitochondrial morphology 25. We therefore utilized transmission electron microscopy (TEM) to assess mitochondrial morphology in AB tumor tissues and healthy control tissues. We found that relative to normal control samples, the numbers of mitochondria were significantly increased from 40.0 ± 2.06 to 62.7 ± 5.48 per 100 μm^2^ but with smaller size in either total mitochondria area percentage or single mitochondria area in AB samples (**Figure 3A**), while the observed mitochondria dynamic changes suggested that these altered mitochondrial dynamics were closely associated with the onset and progression of AB. To modulate Wnt5a protein levels, we next treated AM-1 cells with either recombinant Wnt5a to exogenously up-regulate or with an knockdown adenovirus to down-regulate Wnt5a expression. Wnt5a protein expression was reduced to about 30% of control group after knockdown adenovirus transfection through western blot (**Figure 3B**). These gain- and loss-of-function approaches were then used to assess the influence of Wnt5a on mitochondrial fission and fusion within these AB cells. Through confocal imaging, it was determined that excess Wnt5a resulted in decrease of mitochondrial length, whereas its knockdown had an opposite effect (**Figure 3C**). Consistent with our TEM findings from AB tissues, we additionally found that treatment of AM-1 cells with exogenous Wnt5a was associated with mitochondria dynamic changes (**Figure 3D**). Collectively, our data suggest that both in AB cells or tumor tissues, Wnt5a can regulate dynamics changes of mitochondria.

### Wnt5a modulates mitochondrial-cytoskeletal colocalization via regulating Coronin-1a expression

**Figure 4:**
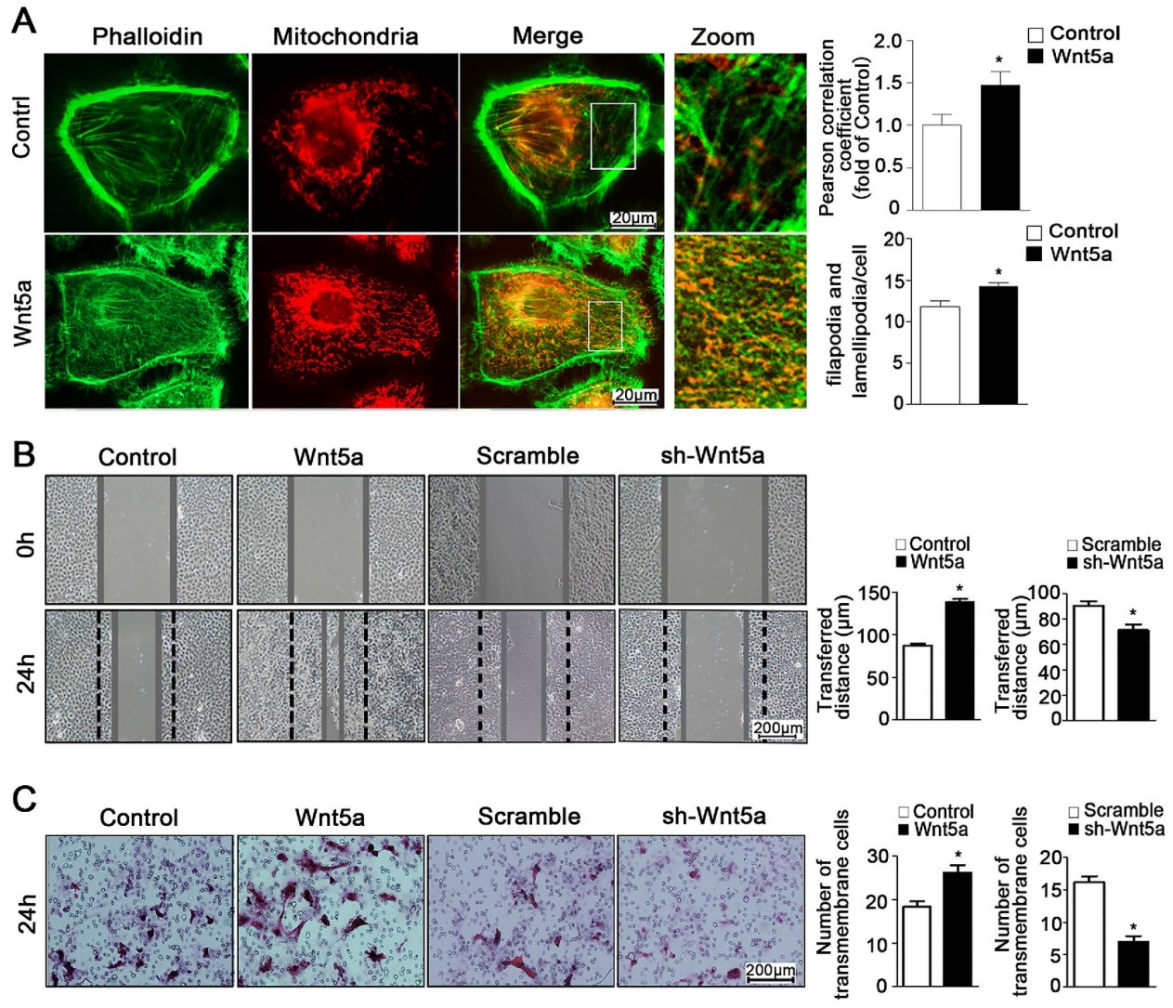
** Wnt5a mediates mitochondria-cytoskeleton morphology and migration behavior in AM-1 cells.** (**A**) Confocal images of AM-1 cell culturing in Wnt-5a recombinant protein and then loaded with FITC-phalloidin and Mito Tracker Red. Scale Bar: 20 µm. Green: FITC-phalloidin, Red: Mito Tracker Red. Pearson correlation coefficient quantification of Mitochondria and cytoskeleton co-localization was shown in bar chart (up). N=25-33 frames from 3 independent experiments. The number of filopodia and lamellipodia was counted and calculated in bar chart (down). N=16-20 cells in each group. (**B**) Scratched at 0h and photographed at 0h, 24h respectively, the transferred distance from Wnt5a up-regulation and down-regulation was calculated and shown in Bar charts respectively. Scale bar: 200 µm, N=9 from 3 independent experiments. (**C**) Transwell assay showing the migration cell numbers of AM-1 cell treated with Wnt5a recombinant protein or sh-Wnt5a adenovirus. Bar charts were the quantitative analysis of up-regulation and down-regulation respectively. Scale bar: 100 µm, N=5. *p<0.05.

Through the use of ultra-high resolution microscopy, it is possible to monitor dynamic changes in inter-organelle contacts and interactions, which play key roles in regulating myriad cellular processes. Importantly for the present study, mitochondria can interact with the cytoskeleton such that mitochondria serve as an energy source that can facilitate cytoskeletal remodeling, while the cytoskeleton itself can regulate changes in mitochondrial dynamics 26, 27. Interplay between these organelles can thus regulate cellular energy availability, intracellular transport, and cell motility 28, 29. In the present study, we found that Wnt5a could increase co-localization between mitochondria and the cytoskeleton with the Pearson correlation coefficient risen from 1.0 ± 0.13 to 1.47 ± 0.16 (**Figure 4A**), thereby modulating cellular morphology and suggesting that the regulation of such contacts may be a key mechanism whereby Wnt5a regulates the behavior of AB cells.

**Figure 5:**
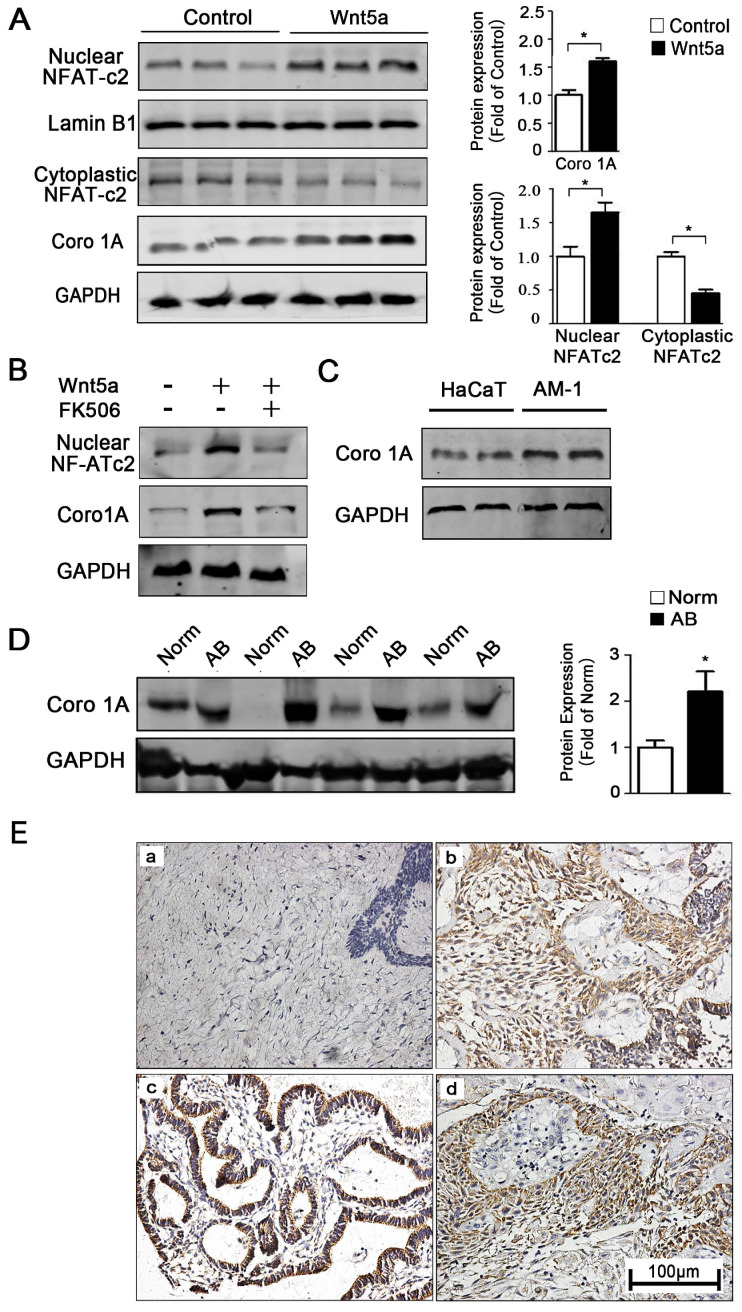
** Wnt5a up-regulates mitochondria-cytoskeleton co-localization through mediating Coro1A expression.** (**A**) Western blot detected nuclear transfactor NFATc2 and Coro1A protein expression. Bar charts demonstrating quantitative significance results from 3 to 5 independent experiments. (**B**) Western blot demonstrating Coro1A protein expression changes of AM-1 cells treated with only human recombinant Wnt5a compared with cells in the presence of both rhWnt5a and FK506 (200 nm). (**C**) Western blot showed Coro1A protein expression in HaCaT and AM-1 cells. (**D**) Western blot results demonstrated Coro1A protein expression in tumor adjacent normal tissues and ameloblastoma tissues. Bar charts showed analysis quantification from 8 pairs of tissues. (**E**) Immunohistochemistry staining demonstrated Coro1A protein expression in normal oral mucosa tissues (a), (b-d) ameloblastoma tissues (forlicular type for b, plexiform type for c, peripheral type for d). Magnification: 200×. *p<0.05.

How precisely Wnt5a regulates mitochondrial functionality remains unclear, and understanding of how interactions between mitochondria and the cytoskeleton are regulated is likewise limited in the context of controlling cellular motility and migration. To further explore how Wnt5a regulates mitochondrial-cytoskeleton interactions, we therefore next conducted gene chip analysis using two pairs of control and recombinant Wnt5a cultivation AM-1 cells (not shown). The results reflected a cytoskeleton binding protein Coronin-1A (Coro1A) with the highest different expression between normal and Wnt5a up-regulated cells. Western blotting confirmed that, relative to control cells, Wnt5a cultivating cells contained 1.65 ± 0.14 folds higher levels of the Coro1A protein (**Figure 5A**). Moreover, Western blot results also showed that more NFAT1 (NFATc2) protein transferred to nuclear (**Figure 5A**). When NFATc2 nuclear translocation inhibitor, FK506, was used to treat Wnt5a cultivating cells, the up-regulation of Coro1A expression induced by Wnt5a was successfully blocked (**Figure 5B**). These results together suggested that such up-regulation of Coro1A may be linked with elevated calcium levels and consequent activation of the NFAT signaling pathway caused by Wnt5a up-regulation. Also, we observed higher Coro1A expression in AM-1 cells relative to levels in control HaCaT cells (**Figure 5C**). We then next assessed Coro1A expression levels via western blotting and immunohistochemistry in 91 AB tissue samples and 20 normal tissue samples (**Figure 5D, E**). These analyses revealed significantly higher rates of Coro1A positivity in AB samples relative to controls (not shown), with this high expression being closely linked to tumor recurrence rate (**Table 2**). Collectively, our data suggest that Wnt5a regulates the mitochondria and cytoskeleton contacts and also up-regulates F-actin binding protein Coro1A expression through activating NFAT signaling pathway.

### Wnt5a-mediated regulation of Coro1A promotes ameloblastoma migration and morphological change

The ability of tumor cells to migrate within an organism is a hallmark of these cells, and is also linked to both ATP production and mitochondrial dynamics 11, 30. As mitochondria move along the cytoskeleton, they can serve as an energy source to support further reorganization of organelles within the cell 26. As such, close contact between the cytoskeleton and mitochondria is a key regulator of the behavior of cells 31, 32. Previous study referred that Wnt5a activation could promote ameloblastoma cell migration 18. Consistently, we found that Wnt5a up-regulation was linked to significant changes of cellular morphology and increased pseudopod formation number from 11.75 ± 0.74 to 14.25 ± 0.43 per cell (**Figure 4A**), and we further found that such exogenous incubation led to significantly increased migration of AM-1 cells (**Figure 4B, C**). Furthermore, we determined that Wnt5a knockdown inhibited AB cell migration (**Figure 4B, C**). Therefore, our results suggest a direct role for Wnt5a in regulating the migratory capacity of these AB cells.

**Figure 6:**
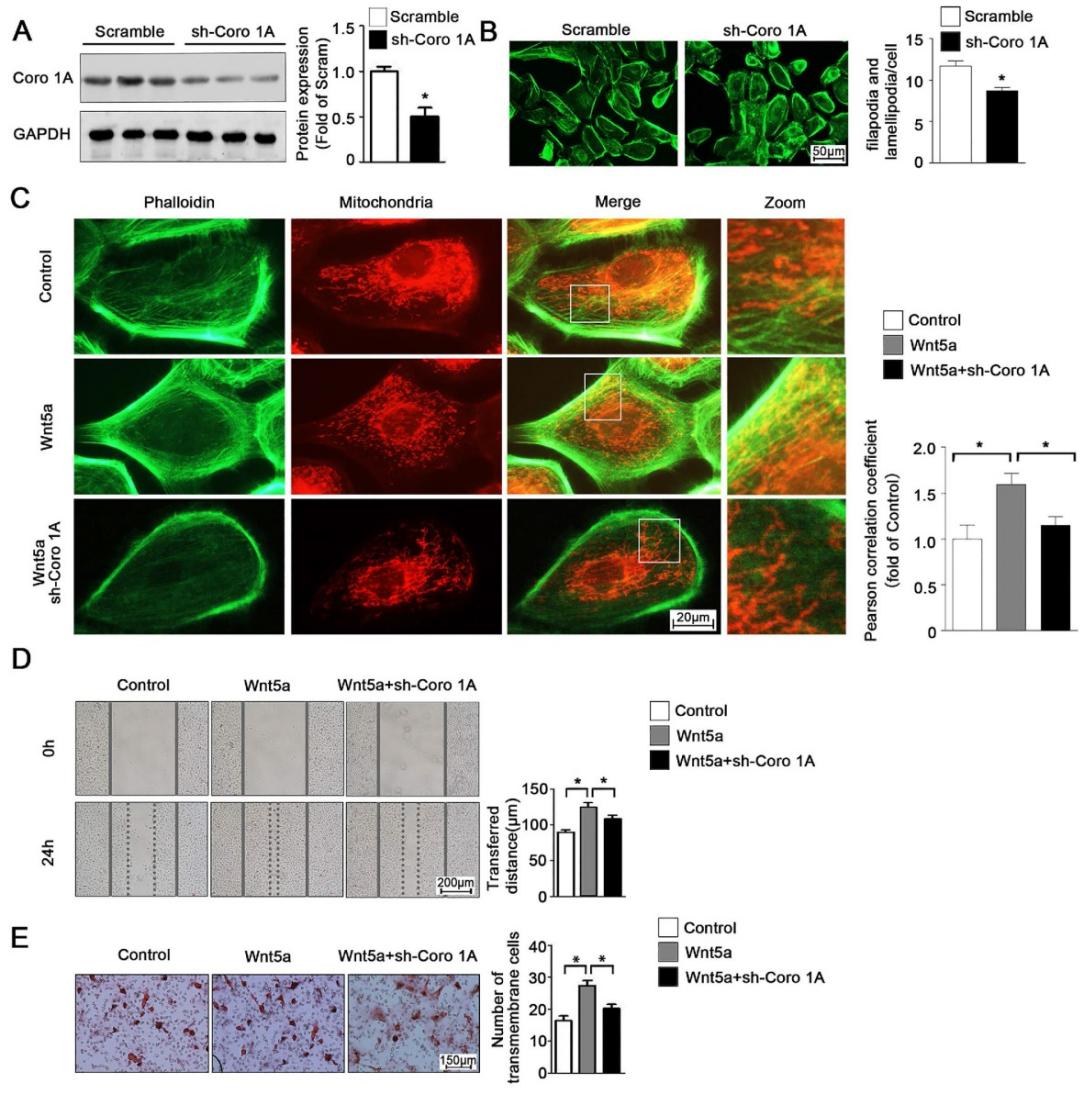
** Coro1A could change F-actin cytoskeleton dynamics and affect AM-1 cell migration.** (**A**) Western blot detecting sh-Coro1A adenovirus transfected efficiency. (**B**) FITC-phalloidin staining showing F-actin and cell morphology changes of both sh-scramble and sh-Coro1A adenovirus transfected AM-1 cells. The number of filopodia and lamellipodia was counted and shown in bar charts, N=18-26 cells in each group. (**C-E**) Sh-scramble and sh-Coro1A adenovirus were transfected for 48-72 h, and then Wnt5a recombinant protein was added. Both Mito tracker Red and FITC-philloidin staining demonstrating mitochondria and cytoskeleton contact changes, and pearson correlation coefficient was counted and shown in bar charts, N=26-43 frames from 3 independent experiments (C); Scratch (D) and transwell assay (E) showing migration ability changes under coro1A decreased expression. N=8 and N=5 independent experiments for scratch and transwell experiments respectively. *p<0.05.

According to the previous studies, Coro1A was considered as an important F-actin binding protein and involved in cytoskeleton dynamic changes. Given this observation, we next sought to determine whether Coro1A played a role in regulating mitochondrial-cytoskeleton interactions or in governing the migration of AB cells through regulation of the cytoskeletal network. To that end, we used adenoviral vectors to generate AM-1 cells in which Coro1A was knocked down (**Figure 6A**). Using these cells, it was determined that Coro1A can regulate cytoskeletal dynamics and pseudopod formation (**Figure 6B**). Moreover, Coro1A knockdown was associated with changes in the mitochondria-cytoskeleton colocalization induced by the addition of recombinant Wnt5a incubation (**Figure 6C**). Finally, Coro1A knockdown was in association with a significant decrease in the migratory ability of AM-1 cells and could partially overturn Wnt5a-induced increase of migration distance from 124.4 ± 6.44 nm to 108.3 ± 5.19 nm (**Figure 6D**) and transmembrane cell number from 27.40 ± 1.63 to 20.20 ± 1.36 (**Figure 6E**). Thus, our results confirm the regulatory role of Coro1A, a Wnt5a downstream targeted protein, on inter-organelle contact networks and AB biological behavior.

## Discussion

In the present study, we offered evidence for the central role of Wnt5a in the regulation of the migration of AB cells owing to its ability to mediate the up-regulation of the F-actin related Coro1A protein and to thereby modulate contact between mitochondria and the cytoskeleton. We found that Wnt5a was significantly up-regulated at the protein level in AB tissue samples relative to normal control tissue samples. In vitro, we further found that Wnt5a up-regulation was associated with increased intracellular calcium levels, in turn leading to changes in mitochondrial dynamics, energy metabolism, and expression of Coro1A. When Wnt5a or Coro1A was knocked down, this reversed these observed changes in intracellular organelle dynamics, and reduced the migratory ability of AB cells.

Previous studies have revealed the elevated Wnt5a expression in more advanced and aggressive disease in patients with melanoma 33, gastric cancer, lung cancer 34 and prostate cancer 35. Furthermore, it has also observed that up-regulation of Wnt5a is associated with changes in the epithelial-mesenchymal transition within AB cells 18, and ours is the first study to report the role of Wnt5a in regulating mitochondrial-cytoskeletal dynamic changes and thereby regulating AB cell migration. Indeed, we observed that Wnt5a was up-regulated in both AB tumor tissue samples and AM-1 cells, consistent with its potential role in the development and progression of AB. The molecular basis for Wnt5a up-regulation in AB will require further research, but may be associated with changes in the expression of certain transcription factors or microRNAs controlling Wnt5a expression 36, 37. Future studies regarding the control of Wnt5a expression in AB and other tumors will be invaluable, as they may offer insight into how to target the Wnt5a signaling pathway to effectively treat these tumors.

The endoplasmic reticulum (ER) serves as a primary calcium storage site within the cell, and can thus release Ca^2+^ into the cytoplasm in specific contexts, resulting in increased local mitochondrial calcium concentrations owing to the close structural interactions between mitochondria and the ER that initiate mitochondrial Ca^2+^ uptake 38, 39. Increases in concentrations of Ca^2+^ within mitochondria can in turn lead to TCA cycle activation and oxidative phosphorylation, thereby enhancing mitochondrial energy production within the cell 25, 40, 41. Importantly, previous work has demonstrated that elevated uptake of Ca^2+^ by mitochondria is frequently associated with the enhanced migration and invasive activity of tumor cells. In this study, we found that Wnt5a can drive elevated intracellular and mitochondrial calcium levels and ATP synthesis, thus potentially providing more energy to facilitate AB cell migration. Elevated mitochondrial Ca^2+^ levels can also drive increased calcium-sensitive dehydrogenase and NADH activity, driving electron flow through the respiratory chain and thereby further increasing ATP synthesis 41. We hypothesized that Wnt5a signaling pathway could result in the elevation of mitochondrial calcium levels within AB cells, thereby driving the enhanced activity of calcium-sensitive enzymes in the respiratory chain and consequently increasing energy production within these tumor cells, thus facilitating their migration ability.

In the present study, we observed changes in the dynamics of mitochondria. A number of different proteins may regulate the fusion and division of mitochondria within cells 42. Our results suggested the possibility that these observed changes in mitochondrial morphology in AB cells may be closely linked to mitochondrial Ca^2+^ homeostasis changes caused by Wnt5a. Such altered calcium homeostasis would in turn could activate PKC signaling and thereby mediate the phosphorylation of fission-associated proteins, thus modulating mitochondrial dynamics 43. The cytoskeleton is a key regulator of the dynamic rearrangement of organelles within cells, allowing for deformation, mitochondrial transport, signaling, and cell motility 44, 45. In this study, we found that Wnt5a could function as a regulator of the rearrangement of this mitochondrial-cytoskeletal network, thereby potentially regulating AB cell migratory activity.

Intracellular calcium signaling can, in addition to the activities outlined above, result in the activation of calcium-sensitive transcription factors 46. These include transcription factors in the NFAT family, which consists of 5 family members, of which four (NFAT1-4) are known to be regulated by Ca^2+^ signaling 47. In the present study, we observed significant NFAT1 activation and nuclear localization in the context of Wnt5a up-regulation, in turn regulating the expression of the downstream target gene encoding Coro1A. Coro1A protein was first shown to be involved in the migratory activity and morphological changes observed in T cells 48, 49. More recent studies have shown Coro1A to be a key F-actin binding protein important for the control of cytoskeletal dynamics in myriad contexts 50, 51. We found that in AB cells, Coro1A regulated cytoskeletal dynamics and interactions between mitochondria and cytoskeleton, thereby regulating the migration of these tumor cells. When Coro1A was knocked down, AB cell migration was significantly reduced, thus identifying Coro1A as a potentially viable therapeutic target for AB treatment. Our results showed that Wnt5a could regulate Ca^2+^ pathway and activate downstream target proteins. However, previous studies also have found that Wnt5a was involved in PCP pathway, which was recognized as an important signaling pathway to regulate tissue polarity and cell migration 52, 53. Since Wnt5a can still promote cell migration in Coro1A-knockdown cells, we hypothesized that other migration related pathways could also be activated by Wnt5a, which still need further investigation.

## Conclusion

Our findings demonstrated the increased Wnt5a expression in AB, and its up-regulation enhanced AB cell migration capacity through increasing intracellular calcium levels, in turn leading to changes in mitochondrial dynamics, energy metabolism, and expression of Coro1A. Knockdown of Wnt5a or downstream Coro1A could reverse these observed changes in intracellular organelle dynamics, and reduce the migratory ability of AB cells. Thus, our findings revealed the role of Wnt5a and Coro1A in AB for the first time, and provided new therapeutic targets for disease progression.

## Figures and Tables

**Table 1:** The differences of Wnt5a expression between ameloblastoma and normal oral mucosal tissues

Groups	Total (N)	Wnt5a Positive (N)	Wnt5a Negative (N)	χ^2^	P
Ameloblastoma	91	78	13	46.70	0.000*
Normal oral mucosal tissue	20	2	18		

P value was determined by Chi-square test. *P<0.05.

**Table 2:** Association between Coro1A expression and clinical pathological features

Characteristics	Total (N)	Coro1A Positive (N, %)	Coro1A Negative (N, %)	χ^2^	P
**Age (year)**				0.080	0.777
≤50	69	40(57.97)	29(42.03)		
>50	22	12(54.55)	10(45.45)		
**Gender**				0.004	0.951
Male	51	29(56.86)	22(43.14)		
Female	40	23(57.50)	17(42.50)		
**Tumor Location**				0.698	0.404
Mandible	80	47(58.75)	33(41.25)		
Maxilla and Gingiva	11	5(45.45)	6(54.55)		
**Pathological type**				0.200	0.655
Solid/Multi-cystic	72	42(58.33)	30(41.67)		
Others (Unicystic/Peripheral/Desmoplastic)	19	10(52.63)	9(47.37)		
**Recurrence**				3.857	0.0495*
Yes	9	8(88.89)	1(11.11)		
No	82	45(54.88)	37(45.12)		

P value was determined by Chi-square test. *P<0.05.

## Abbreviations

AB: ameloblastoma
NOM: normal oral mucosa
TEM: transmission electron microscopy
Coro1A: Coronin-1A
ER: endoplasmic reticulum
